# Structure, Solubility and Stability of Orbifloxacin Crystal Forms: Hemihydrate *versus* Anhydrate

**DOI:** 10.3390/molecules21030328

**Published:** 2016-03-09

**Authors:** Olimpia Maria Martins Santos, Jennifer Tavares Jacon Freitas, Edith Cristina Laignier Cazedey, Magali Benjamim de Araújo, Antonio Carlos Doriguetto

**Affiliations:** 1 Institute of Chemistry, Federal University of Alfenas, Rua Gabriel Monteiro da Silva, 700, Alfenas-MG 37130-000, Brazil; olimpia_martins@yahoo.com.br; 2 Faculty of Pharmaceutical Sciences, Federal University of Alfenas; Rua Gabriel Monteiro da Silva, 700, Alfenas-MG 37130-000, Brazil; jennifer_jacon@yahoo.com.br (J.T.J.F); crislaignier@yahoo.com.br (E.C.L.C.); magali@unifal-mg.edu.br (M.B.d.A.); 3 Faculty of Pharmacy, Federal University of Bahia, Campus Ondina, Rua Barão de Jeremoabo, 147, Ondina, Salvador-BA 40170-115, Brazil

**Keywords:** orbifloxacin, crystalline structure, solubility, stability

## Abstract

Orbifloxacin (ORBI) is a widely used antimicrobial drug of the fluoroquinolone class. In the official pharmaceutical compendia the existence of polymorphism in this active pharmaceutical ingredient (API) is reported. No crystal structure has been reported for this API and as described in the literature, its solubility is very controversial. Considering that different solid forms of the same API may have different physicochemical properties, these different solubilities may have resulted from analyses inadvertently carried out on different polymorphs. The solubility is the most critical property because it can affect the bioavailability and may compromise the quality of a drug product. The crystalline structure of ORBI determined by SCXRD is reported here for the first time. The structural analysis reveals that the ORBI molecule is zwitterionic and hemihydrated. ORBI hemihydrated form was characterized by the following techniques: TG/DTA, FTIR-ATR, and PXRD. A second crystalline ORBI form is also reported: the ORBI anhydrous form was obtained by heating the hemihydrate. These ORBI solid forms were isomorphous, since no significant change in unit cell and space group symmetry were observed. The solid-state phase transformation between these forms is discussed and the equilibrium solubility data were examined in order to check the impact of the differences observed in their crystalline structures.

## 1. Introduction

Fluoroquinolones are a class of antimicrobials that are widely used in human and veterinary medicine [1,2,3]. This group of antimicrobial agents has significant bactericidal activity against a wide range of veterinary pathogens and Gram-negative bacteria (*Escherichia coli*, certain species of *Enterobacter*, *Klebsiella*, *Pasteurella*, *Proteus*, *Salmonella*, and *Pseudomonas*) and some Gram-positive bacteria, such as *Staphylococcus* spp., *Chlamydia*, *Mycobacteria*, *Mycoplasma*, and *Ureaplasma* spp. [2,4].

**Figure 1:**
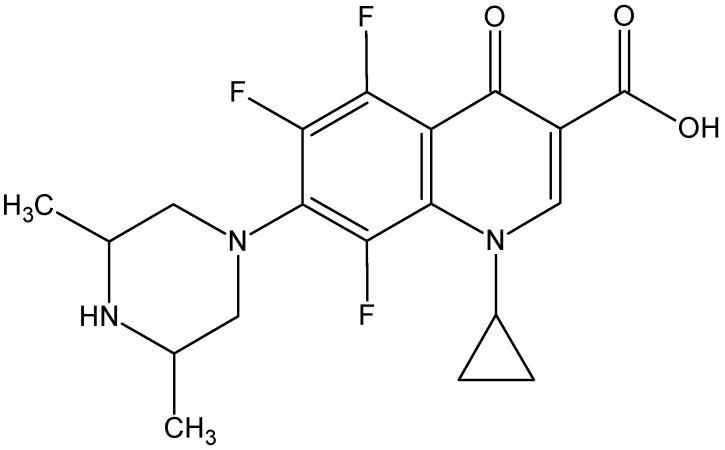
Chemical structure of the antimicrobial drug orbifloxacin.

Orbifloxacin (ORBI, 1-cyclopropyl-7-[(3*S*,5*R*)-3,5-dimethylpiperazin-1-yl]-5,6,8-trifluoro-4-oxo-1,4-dihydroquinoline-3-carboxylic acid, Figure 1), is a third-generation synthetic fluoroquinolone that has been developed exclusively for the treatment of gastrointestinal and respiratory infections in animals [1,5,6].

According to the Merck Index, ORBI is a slightly yellow crystalline powder that is freely soluble in water, soluble in methanol, and slightly soluble in ethanol, ether, ethyl acetate, and chloroform [7]. However, in the British and US Pharmacopoeias, ORBI appears as white crystals or pale yellow crystalline powder that is very slightly soluble in water, methanol, and chloroform; soluble in glacial acetic acid; and practically insoluble in anhydrous ethanol [8,9].

This solubility was recorded at a temperature between 15 °C and 25 °C according to the pharmacopeias. “Freely soluble in water” [7] means that 1 g of ORBI is soluble in 1–10 mL of solvent; thus, between 0.1 and 1 g of ORBI is soluble in 1 mL of water. In contrast, “very slightly soluble” [8,9] means that 1 g of ORBI is soluble in 1000–10,000 mL of solvent; thus, between 0.0001 and 0.001 g of ORBI is soluble in 1 mL of water [8,9]. This discrepancy can be attributed to crystalline solid state changes, since the British and U.S. Pharmacopoeias report the existence of polymorphism in this API.

Polymorphs are crystals that have the same chemical composition but are arranged in at least two different forms in the solid state [10,11,12]. However, in pharmaceutical sciences the term is used with a wider range of species that also includes other solid mono- and multicomponent forms of APIs and excipients such as hydrates, solvates, amorphous, salts and co-crystals [13].

Drug polymorphism is a phenomenon known since the 1960s, when Aguiar and colleagues analysed two solid forms of the antibiotic chloramphenicol palmitate. It was concluded that forms A and B of chloramphenicol palmitate showed different dissolution rates and different blood levels, and only form B showed adequate bioavailability for the expected therapeutic effect [14].

Thus, the different solubilities reported for ORBI may have resulted from an inadvertent analysis of different polymorphs in the powdered material. Solid forms of the same API may have different physicochemical properties [10,14,15]. The different intermolecular interactions among the various existing solid forms of an API can generate these differences. The solubility is the most critical property because it can affect the bioavailability and may compromise the development of a bioequivalent product [10,15,16,17]. The Food and Drug Administration (FDA) [18] and the International Conference on Harmonization (ICH) [19] require preliminary and exhaustive screening studies to identify and characterize all the polymorphic forms for each drug to avoid this problem [20].

Therefore, because the polymorphism phenomenon is probable to occur for ORBI, it is very important to have access to the crystal structures of their different polymorphs and to study whether there are differences in their solubilities and other properties. These types of studies can avoid problems such as toxicity or therapeutic failure [10,12,21].

Single-crystal and powder X-ray diffraction techniques are the most suitable and commonly utilized tools to study and characterize polymorphs in pharmaceutical solids because they provide unequivocal proof of polymorphism existence [18]. Following our ongoing research to determine the crystal structures of APIs [21,22,23,24,25,26,27,28,29,30], we report here for the first time two crystalline structures of ORBI. The first ORBI crystal structure, which was determined by single-crystal X-ray diffraction, shows the ORBI molecule in a zwitterionic form. Because the crystal is a hemihydrate, this structure is termed the ORBI hemihydrate form. ORBI hemihydrate was characterized by thermal analysis (TG/DTA), infrared spectroscopy, and powder X-ray diffraction. A second crystalline form, the ORBI anhydrous form obtained from the hemihydrate form by heating it, is also reported. The solid-state phase transformation between the ORBI anhydrous and hemihydrate forms during wetting and drying is presented. The equilibrium solubility of the hemihydrate and anhydrous forms were compared after 48 h (at 150 rpm and 25 °C) in a physiological pH range (~1 to 7.5) to assess the impact of the crystal structure differences on solubility.

## 2. Results and Discussions

### 2.1. Structure Determination by Single-Crystal X-ray Diffraction

Crystal data, data collection procedures, structure determination methods, and refinement results for ORBI hemihydrate form are summarized in Table 1.

**Figure 2:**
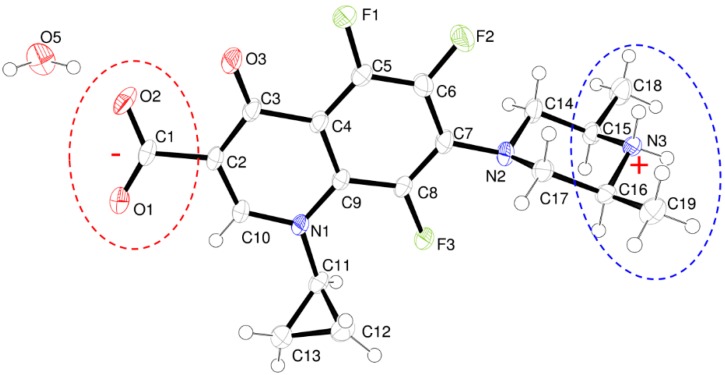
ORTEP view of ORBI hemihydrate form showing the atoms displacement ellipsoids at 50% probability level. Non-hydrogen atoms are arbitrarily labelled and hydrogen atoms are shown as spheres of arbitrary radii. The protonated and deprotonated moieties are marked by dotted circles in blue and red, respectively. The molecules with C15(R),C16(S) configuration was arbitrarily chosen.

**Figure 3:**
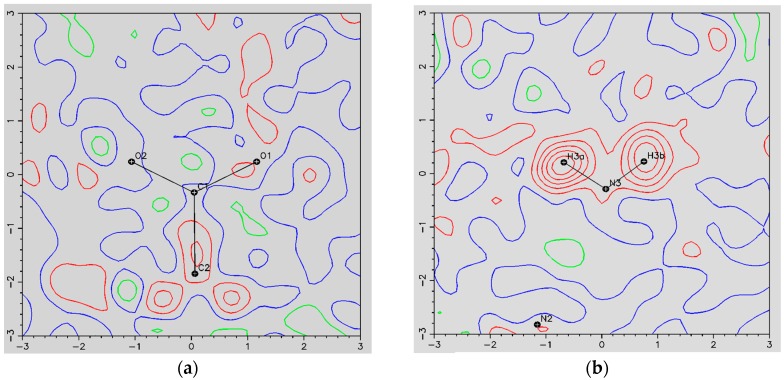
Residual density maps through the deprotonated (carboxylate) (**a**) and protonated (piperazine) (**b**) moieties from ORBI hemihydrate form, obtained by difference Fourier synthesis. Red, green and blue lines show positive, negative, and zero contours levels, respectively. Contour levels correspond to 0.1 e·Å^−3^ intervals.

Figure 2 is an ORTEP-3 view showing the ORBI hemihydrate form asymmetric unit. Three important structural features should be highlighted before analysing its intra- and intermolecular geometries:

(a) The structure is a hemihydrate (one half water molecule for each ORBI molecule), and the water oxygen atom lies in a special position (lies in the 2-fold axis parallel to the unit cell *a* axis) of the C222_1_ space group. Therefore, the two water hydrogen atoms shown in Figure 2 are crystallographic dependent by symmetry.
(b) The ORBI molecule is in its zwitterion form with the carboxylic hydrogen transferred to pyperazine nitrogen. The proton transfer was confirmed by Fourier difference maps, which show the absence of residual electron density close to the oxygen atoms of the carboxylic group and the presence of two large peaks of electronic density close to the pyperazine nitrogen, which clearly reveals the presence of two hydrogens linked to it (Figure 3). Moreover, identical bond lengths are observed for C1-O1 (1.250(3) Å) and C1-O2 (1.251(2) Å), indicating the formation of a carboxylate ion (negative charge resulting from deprotonation is delocalized between the two oxygen atoms).
(c) Since ORBI molecule has two stereocenters (at C15 and C16) and it is crystallized in a non-centrosymmetric space group (Table 1) containing just one molecule in the asymmetric unit, its crystal structure is expected to contain a pure enantiomer [31]. However, this is not the case because the piperazine ring containing the two stereocenters has as internal mirror plane (through N2, N3, and the hydrogens linked to N3) that bisects this moiety. The symmetrical nature arising from the presence of two stereogenic centres with identical substitution but opposite configuration makes ORBI a *meso*-compound. As highlighted by Hoffmann, *meso*-compounds can reach the same conformation with a plane of symmetry or a centre of inversion among the continuum of freely accessible conformations [32]. The ORBI C15(R),C16(S) and C15(S),C16(R) isomers are equivalent (superimposable on its internal mirror image) considering the pyperazine moiety. Additionally, because the quinolone and pyperazine rings can freely rotate around the C7-N2 bond, the whole molecules containing the two isomers are also equivalent. ORBI is therefore optically inactive; the contributions of the two stereogenic centres to the Cotton effect compensate for each other [32]. Therefore, the C15(R),C16(S) isomer shown in Figure 2 was arbitrarily chosen.

For the ORBI hemihydrate form, it is observed that its quinolone ring is, as expected, very planar. The root mean square deviation (r.m.s.d) of the least-square plane thought the two fused-rings atoms is 0.0623. The largest deviations from the calculated least-square plane considering the first neighbour atoms linked to the quinolone ring occur for O3 (−0.289(3) Å) and C1 (0.295(3) Å). The carboxylate oxygens also deviate significantly from the quinolone ring (0.167(3) and 0.638(3) Å for O1 and O2, respectively). In spite of the overall planar character, there is a slight bend in the quinolone ring along an imaginary line passing through C3 and N1. The least-square plane through the moiety containing C2, C3, C10, and N1 (r.m.s.d = 0.0062) makes an angle of 10.30(7)° relative to the plane calculated through the remaining quinolone two-fused ring including C3 and N1 (r.m.s.d = 0.0090).

The piperazine ring has a chair conformation, which was confirmed by the Evans and Boeyens conformational analysis [33]. The N2-C7, C15-C18, C16-C19, C14-H14B, N3-H3b, and C17-H17a bonds are in the equatorial positions, whereas the remaining bonds are axial.

The molecular conformation was analysed using MOGUL [34], a knowledge base derived from the Cambridge Structural Database, CSD [35], which provides quick access to information on the preferred length, angle, and torsion values of the chemical bonds in the structures. This comparative study of the intramolecular geometry of unpublished crystal structures similar to ones reported in the CSD is useful to check for errors in the refinement or to probe unusual geometries [34]. In the case of ORBI, the study showed that all the bond lengths, bond angles, and torsion angles are in agreement with the expected values.

**Figure 4:**
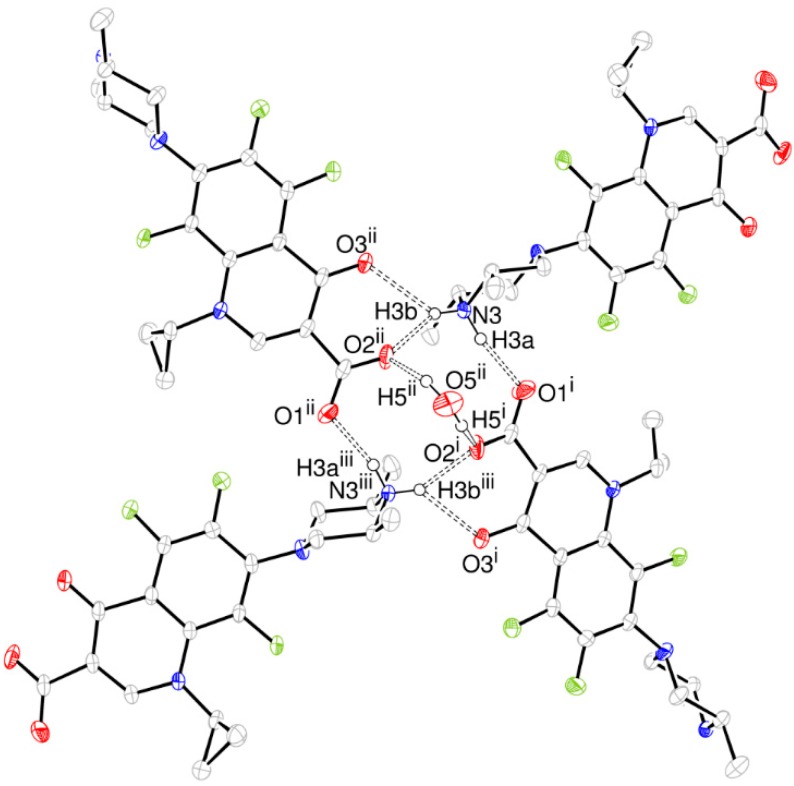
ORTEP-3 representation of the ORBI tetramer stabilized by hydrogen bonds (dashed line) viewed normal to the unit cell *a* axis. The hydrogen atoms not involved in the depicted hydrogen bonds were omitted for clarity. Symmetry codes: ^i^ –x + 1/2, y-1/2, –z + 3/2; ^ii^ –x + 1/2, –y + 1/2, z + 1/2; ^iii^ x, –y, –z + 2.

Taking into account the intermolecular interactions, a tetramer assembled through intermolecular H bonds from molecules related to 2_1_- and 2-fold symmetry axis is the building block for the ORBI hemihydrate form (Figure 4). This supramolecular synthon involves four ORBI molecules diametrically oriented (tetramer) with one pair of molecules having the piperazine extremity facing the ring centre and another pair facing the carboxylate (Figure 4).

The piperazine nitrogen (N3) acts through H3a as a hydrogen bond donor to O1, and through H3b as a bifurcated hydrogen bond donor to O2 and O3. Both water hydrogen atoms are hydrogen bonded to two diametrical ORBI molecules through their O2 carboxylate oxygens. However, the water oxygen does not act as hydrogen bond acceptor for any atom in this structure. The hydrogen bond geometry involved in the packing is given in Table 2.

**Figure 5:**
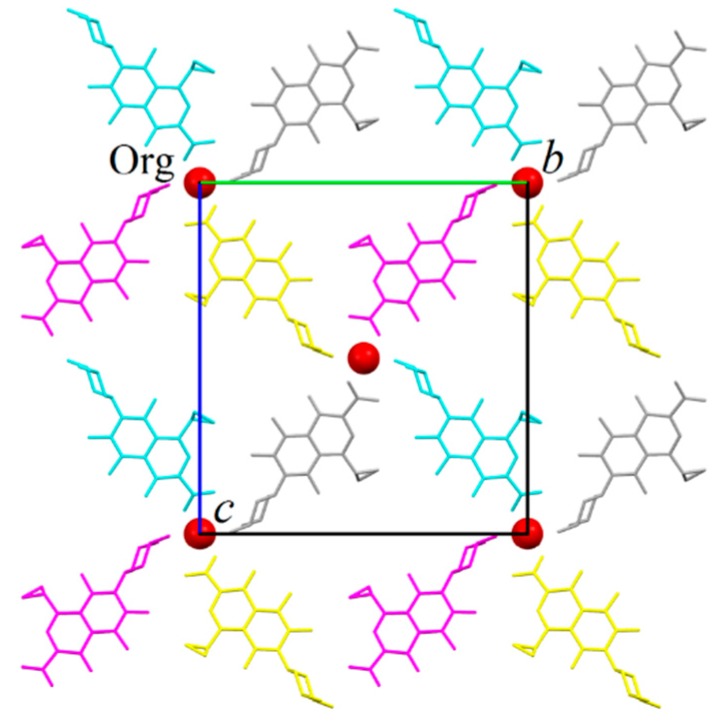
Partial packing of ORBI hemihydrate form showing a layer formed normal to the unit cell *a* axis. The layer was generated from the asymmetric unit applying the symmetry operations included in Figure 4 captions. The colour scheme refers to four of the eight equivalents by symmetry positions of the C222_1_ space group. Water oxygen atoms are represented as red spheres. Hydrogens were omitted for clarity.

Each tetramer is surrounded by four others by sharing its ORBI molecules, forming a 2D infinite network (layer) parallel to the (100) plane (Figure 5). The red spheres representing the water molecules in Figure 5 coincide with the tetramer linkage points. All linkage points contain the hydrogen bonds discussed above for the tetramer shown in Figure 4.

The water molecules are connected to the layer by the hydrogen bond involving the O2 carboxylate oxygen (see Figure 4). The layer stacking is governed by the 2_1_-fold screw axis parallel to the unit cell *c* axis. Therefore, the layers stacked along the [100] direction alternate a translation of *c*/2 (or *b*/2) one over the other. Thus, the tetramer centre localized at (x, 0, 0), (x, 1, 0), (x, 0, 1), (x, 1, 1), and (x, ½, ½) positions in the layer shown in Figure 5 will be displaced to the (x, 0, ½) (x, ½, 0), (x, 1, ½), and (x, ½, 1) positions for the layer stacked below or above it.

It is clearly observed that the crystallization waters occupy channels parallel to the unit cell *a* axis and coincident with the positions of the tetramers’ centres (also tetramer linkage points). Because the water oxygen is in the special crystallographic position of the C222_1_ space group (fraction coordinate x, ½, ½), the channels lies, of course, in the 2-fold axis parallel to the unit cell *a* axis. Later, it will be discussed that this strongly layered supramolecular structure is maintained even without the presence of water (ORBI anhydrous) in the channel, and therefore, ORBI hemihydrate/anhydrous structures present zeolite behaviour.

**Figure 6:**
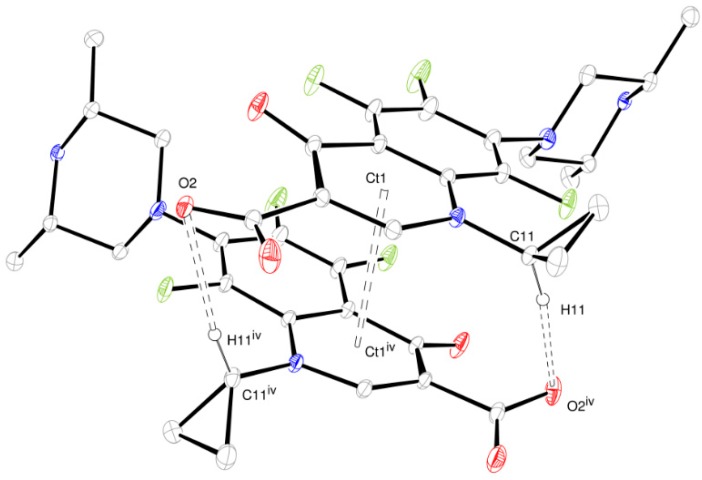
ORTEP-3 view of the inter-layer intermolecular contacts stabilized by non-classical hydrogen bonds and π-aryl-π-aryl interactions (dashed line). Ct is the aromatic ring calculated centroid. The hydrogen atoms not involved in the depicted hydrogen bonds were omitted for clarity. Symmetry code: ^iv^ −x, y, −z + 3/2.

The layer stacking is stabilized by non-classical hydrogen bonds involving inter-layer ORBI molecules. The propyl cycle methine hydrogen works as a hydrogen bond donor to O2 (Figure 6). Therefore, O2 is a trifurcated hydrogen bond acceptor because it is also an acceptor from the water oxygen and piperazine nitrogen (Figure 4). π-aryl-π-aryl interactions with centroids (Ct1…Ct1^iv^, iv = −x, y, −z + 3/2) separated by 3.580 Å also help in the inter-layer stabilization.

### 2.2. ORBI Characterization

#### 2.2.1. Powder X-ray Diffraction

**Figure 7:**
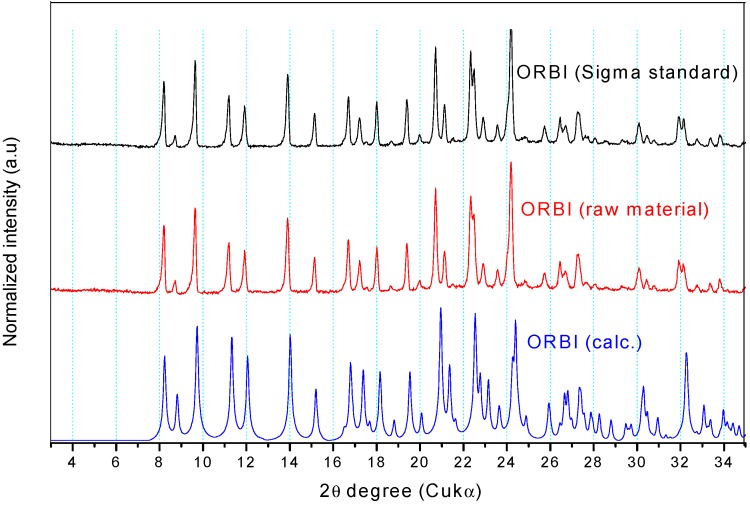
Experimental and calculated PXRD patterns of ORBI. The calculated pattern is in blue, and the experimental patterns for the raw material and the Sigma standard are in red and black, respectively.

Figure 7 shows that the experimental Powder X-ray Diffraction (PXRD) patterns of the ORBI standard (Sigma) and the ORBI raw material are matched, indicating that the two ground materials crystallize in the same crystal phase. Moreover, both patterns match the calculated PXRD of the ORBI hemihydrate form for which crystal structure was determined herein.

Because there were no pronounced broad humps from amorphous solids or extra Bragg reflections from crystalline phases other than that of the ORBI hemihydrate form, upon observing the experimental patterns it was possible to say that the Sigma ORBI standard and the ORBI raw material are formed in bulk by the hemihydrate phase of ORBI determined here.

#### 2.2.2. Thermal Analysis

**Figure 8:**
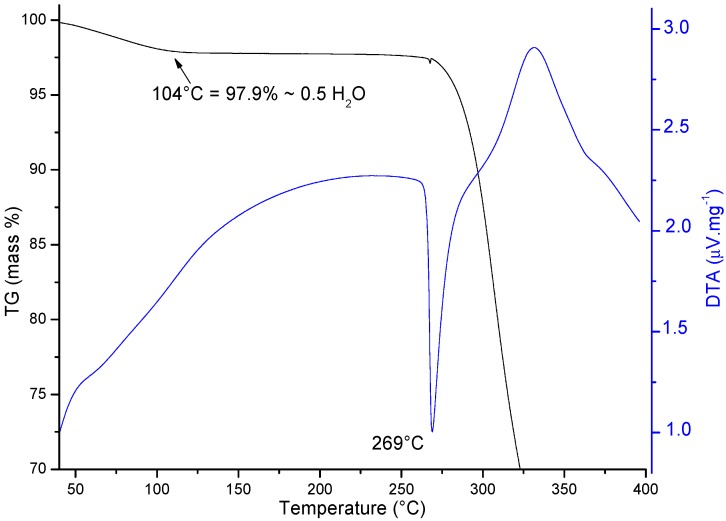
TG (in black) and DTA (in blue) curves measured from 40 to 400 °C starting with ORBI hemihydrate. The arrow shows the temperature of the complete water loss from the structure.

The TG/DTA curves recorded for the Sigma ORBI standard show, as expected, an initial mass loss corresponding to the dehydration (0.5 mol of water per mol of ORBI) of the sample that was shown to be a hemihydrate by the diffraction techniques (Ti = 75 °C; Tf = 104 °C; Δm_TG_ = 2.1%/Δm_calc_. = 2.23%) (Figure 8). Therefore, the molar ratio of 1:2 for water:ORBI was confirmed in the raw material by TG/DTA analysis. The dehydrated product (ORBI anhydrous) is stable up to ~260 °C, at which point its thermal decomposition begins (DTA peak at 269 °C).

#### 2.2.3. Infrared Spectroscopy with Attenuated Total Reflectance by Fourier Transform (FTIR-ATR)

Considering that TG/DTA results obtained here indicated two different crystalline phases of ORBI (hemihydrate and anhydrous), FTIR-ATR data were measured for the Sigma ORBI standard samples without (as received) and with *ex-situ* thermal treatment (obtained by heating 10 mg of Sigma ORBI standard sample for 2 h at 105 °C) (Figure S1—Supplementary Materials).

The characteristic bands of ORBI were assigned based on that one’s attributed to similar quinolones [36]. The band in the region of 3446 cm^−1^ refers to the -NH vibrations of the piperazinic ring, whereas the bands between 3018 and 2763 cm ^−1^ can be assigned to axial deformations of the C-N bond of piperazine. The intense band in the region of 1700 cm^−1^ corresponds to the C=O group of the carboxylic acid. The region from 1050 to 1000 cm^−1^ shows vibrations related to axial deformations of C–F groups. Vibrations below 1000 cm^−1^ refer to the angular deformations out of plane of aromatic –CH.

The unique significant difference between the two spectra occurs at approximately 3340 cm^−1^, which is the region of water axial deformation. As expected, an attenuation of this broad band for the ORBI anhydrous form was observed.

### 2.3. Interconversion Study between ORBI Anhydrous and Hemihydrate Forms

The TG curves of the Sigma ORBI standard as received (ORBI hemihydrate form), ORBI standard after heating (ORBI anhydrous form), and ORBI Standard after heating followed by treatment in a climatic chamber (rehydrated form) were compared (Figure S2—Supplementary Materials). The TG curve of the *ex-situ* heated sample at 200 °C for 10 h does not show mass loss in the range observed for the ORBI hemihydrate form. Therefore, the ORBI anhydrous form can be obtained, as expected, by heating the hemihydrate form in the 104–269 °C range. Interestingly, the TG curve of a previously dehydrated ORBI sample introduced in the climatic chamber at 40 °C and 75% relative humidity for 1 h match that of the Sigma ORBI Standard (hemihydrate form). Therefore, the ORBI anhydrous form can be easily returned to the hemihydrate one by exposing the former to high temperature/humidity conditions.

**Figure 9:**
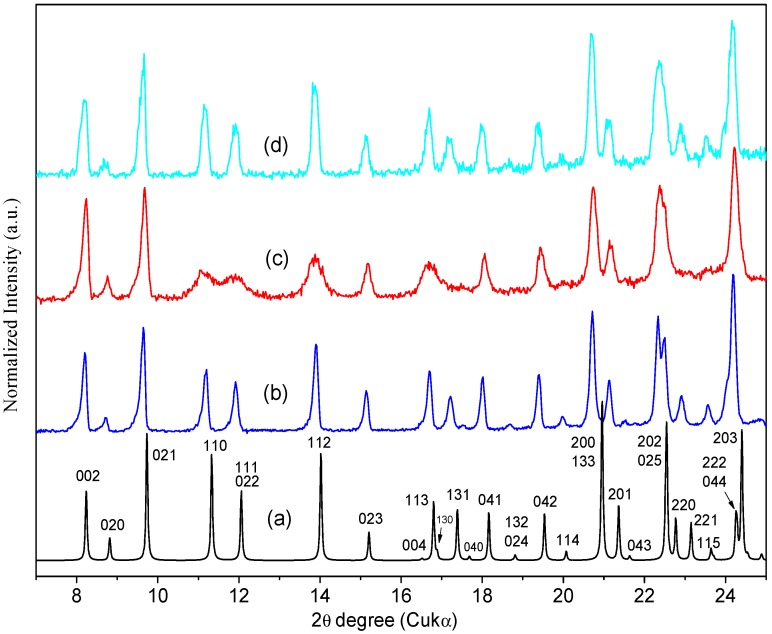
PXRD patterns of ORBI: (a) Calculated from the ORBI hemihydrate form (the Miller indices identifying the Bragg peaks are included); (b) experimental PXRD patterns of Sigma ORBI Standard as received (ORBI hemihydrate form); (c) experimental Sigma ORBI Standard after heating at 200 °C for 10 h (ORBI anhydrous form); (d) Sigma ORBI Standard after heating at 200 °C for 10 h followed by treatment in a climatic chamber at 40 °C with 75% relative humidity during 1 h (rehydrated form = ORBI hemihydrate form). All thermal treatments were *ex-situ*.

The experimental PXRD pattern of the sample heated *ex-situ* at 200 °C retains the Bragg peaks of the ORBI hemihydrate form (Figure 9c). This result confirms that no crystallographic phase transition occurs when the ORBI hemihydrate form has its structural water released to generate the ORBI anhydrous form. Thus, the hemihydrate and anhydrous forms are isomorphous crystals [37].

Although it does not elicit a crystallographic phase transition, the water release causes structural distortions. An evident peak broadening is observed for some hkl Bragg peaks. Specifically, the hkl Bragg peaks with h ≠ 0 (e.g., 110, 111, 112, 113) are broader than those with h = 0 (e.g., 002, 021, 020, 023), suggesting that ORBI hemihydrate form dehydration is followed by an anisotropic loss of crystallinity along the unit cell *a* axis.

The dehydration process keeping the crystal structure can be explained considering the packing of the ORBI hemihydrate form. It should be remembered that the water molecules of the ORBI hemihydrate form lie in the channels and are linked to the ORBI molecules only as hydrogen bond donors. These structural features in addition to the very stable intralayer structures formed by supramolecular tetramers of ORBI molecules are probably responsible for the ORBI water desorption not disrupting the crystal structure. It is important to emphasize that the void volume occupied by the water molecule in the ORBI hemihydrate form is 1.7% (calculated by MERCURY (see Experimental Section 3.3) using a grid spacing and a probing-sphere radius of 0.7 and 1.1 Å, respectively). It is normal that channel solvates with void volumes less than 10% do not collapse on solvent removal [38].

The intralayer stability explains the small widening of the Bragg peaks with 0kl indices observed in the experimental PXRD pattern of the ORBI anhydrous form (Figure 9c). The water presence appears to be more important to the interlayer stability, working as an intercalation guest in the layered ORBI host structure. This explains the preferential broadening of the Bragg peaks depending on the unit cell *a* axis (hkl indices with h ≠ 0).

The PXRD analysis corroborates the hydration of the ORBI anhydrous form in the climatic chamber. Moreover, they show that rehydrated sample returns to the ORBI hemihydrate form. The broad peaks observed in the PXRD pattern of the anhydrous sample become narrow again in the PXRD pattern of the rehydrated sample. Thus, the ORBI porous structure also favours the inverse process of water adsorption not disrupting the crystal structure. Therefore, the ORBI crystal structure can be considered a nanoporous API with adsorption/desorption features.

It is important to emphasize that water adsorption/desorption of crystalline materials may be of three types; the crystal structure can undergo no or little change, a reversible change, or an irreversible change. Water adsorption/desorption without crystal structure changes is well reported for zeolite materials [39]. Similarly, desolvated and solvated APIs may have the same crystal structure with relatively small changes in cell parameters and atomic coordinates [40]. When the structure is maintained during the water adsorption/desorption processes, it is usually more difficult to distinguish between hydrated and anhydrous forms by PXRD [40]. Fortunately, the hemihydrated and anhydrous forms of ORBI can be distinguished here by PXRD by comparing their Bragg peak widths (hkl indices with h ≠ 0).

### 2.4. Characterization of ORBI Tablets

ORBAX™ tablets were ground and analysed by PXRD (Figure S3—Supplementary Materials). Although the excipient amount (mass) is higher than the API mass in the studied tablets (the average weight (*n* = 20 tablets) was 296.7 mg, *i.e.*, 22.7 mg of orbifloxacin and 274 mg of excipient) and it contains crystalline compounds such as lactose monohydrate, it was possible to identify the Bragg peaks of the ORBI structure determined here. Although the Bragg peaks with hkl indices with h ≠ 0 appear to have a width compatible with the ORBI hemihydrate form, the presence of the phases corresponding to excipient preclude the definitive assignment of the API as hemihydrate or anhydrous forms or a mixture of the two in the tablets.

Unfortunately, the TG analysis and FTIR spectroscopy, which were useful to distinguish between the two ORBI forms in the ORBI raw material, could not be applied in the ORBAX™ case due to the relative amount of ORBI compared to the excipient and the presence of hydrated crystalline components in excipients (e.g., lactose monohydrate). In TG curves for example, the calculated percentage of water loss from the ORBI in the tablets (excipient + API) is only 0.16%, which is very difficult to detect confidently by this technique.

### 2.5. Solubility Studies of Hemihydrated and Anhydrous Forms of ORBI

**Scheme 1:**
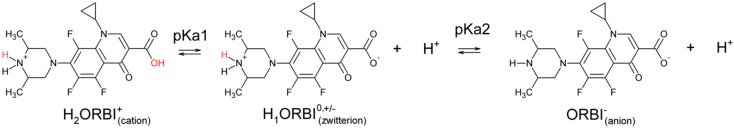
ORBI acid dissociation constants.

The ORBI molecule has two acid dissociation constants: pKa1 = 5.6 for the 3-carboxylic group, and pKa2 = 8.9 for the 3,5-dimethylpiperazinyl group [7], as shown in Scheme 1. As such, three pH-dependent acid-base species are possible in aqueous media, and depending on the pH, ORBI can be crystallized as a neutral zwitterionic form or as anionic or cationic salts.

**Figure 10:**
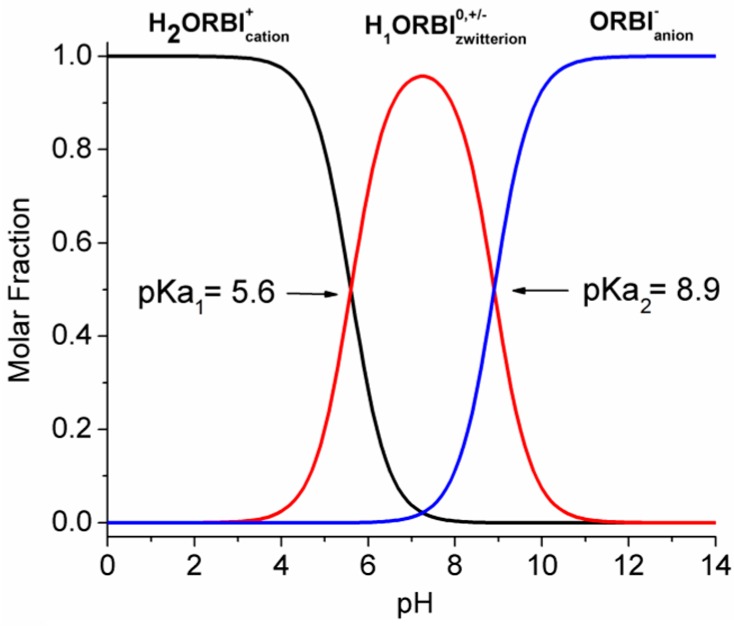
pH-dependent acid-basic ORBI species distribution. The molar fraction (α) of each species was calculated as follows: $\alpha_{2}=\left[H_{2}ORBI_{\left(cation\right)}^{+}\right]/C\;\;=\left(1+(Ka_{1}/\left[H^{+}\right]\right)\;+\;(Ka_{1}\times Ka_{2}/{\left[H^{+}\right]}^{2}\;){)}^{-1}$; $\alpha_{1}=\left[H_{1}ORBI_{\left(zwitterion\right)}^{0,+/-}\right]/C\;\;=\alpha_{2}(Ka_{1}/\left[H^{+}\right])$; $\alpha_{0}=\left[ORBI_{\left(anion\right)}^{-}\right]/C\;=\alpha_{2}\left(Ka_{1}\times Ka_{1}/{\left[H^{+}\right]}^{2}\right)$; where $\alpha_{0}+\alpha_{1}+\alpha_{2}=1$ and $C=\left[H_{2}ORBI_{\left(cation\right)}^{+}\right]+\left[H_{1}ORBI_{\left(zwitterion\right)}^{0,+/-}\right]+\left[ORBI_{\left(anion\right)}^{-}\right]$ . Curves show pH in steps of 0.1.

It is important to emphasize that the ORBI molecule is zwitterionic in the two solid-state forms described in this work (hemihydrate and anhydrous forms). Additionally, considering the pH-dependent acid-basic ORBI species distribution, the zwitterionic ($H_{1}ORBI_{\left(\text{zwitterion}\right)}^{0,+/-}$) species is expected to be the predominant one in the pH range between pKa1 and pKa2 (Figure 10).

**Figure 11:**
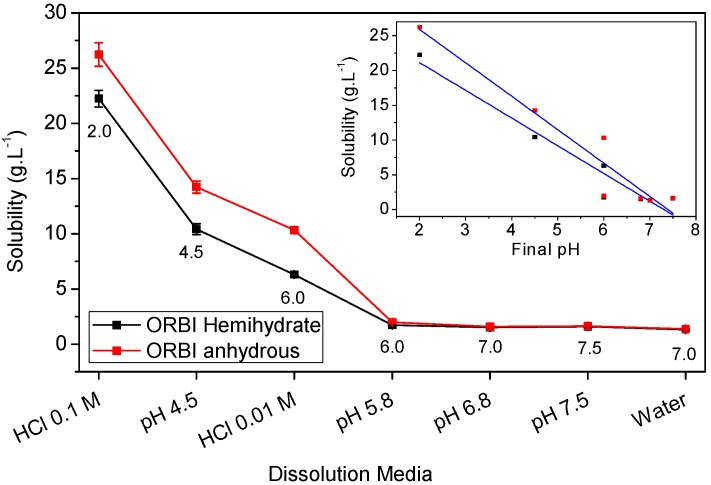
Equilibrium solubility values for ORBI anhydrous and hemihydrate forms at 25 °C, 150 rpm, 48 h. Error bars represent standard deviations (among 0.039 and 0.751 g·L^−1^, *n* = 3). The inset shows the correlation including the linear regression (blue lines) between the equilibrium solubility values with the final pH of the different used media for the ORBI hemihydrate and anhydrous forms.

Therefore, the ORBI hemihydrate and anhydrous forms’ solubility in a neutral pH range (zwitterion prevalence) is expected to be lower than that at either acid or alkaline pH (cationic or anionic prevalence) extremes. In accord with these expectations, the lowest values of equilibrium solubility were observed in the neutral media, including water (pH = 7) and pH 5.8, 6.8, and 7.5 buffers, for both the hemihydrate and anhydrous ORBI forms (Table 3 and Figure 11).

Also as expected, considering the pH-dependent acid-basic ORBI species distribution in solution, the equilibrium solubility values of both ORBI crystal forms increase significantly in aqueous hydrochloric acid and in pH 4.5 buffered media. For instance, the equilibrium solubility of the anhydrous ORBI form is 19-fold greater in HCl 0.1 mol·L^−1^ than in water (Table 3 and Figure 11).

Moreover, Figure 11 shows that there is a linear correlation of the equilibrium solubility for both forms with the final pH of the used media. The pH-solubility profile for the two ORBI forms studied here increasing as the pH decreases is due to the protonation ($H_{1}ORBI_{\left(\text{zwitterion}\right)}^{0,+/-}$ + H^+^ → $H_{2}ORBI_{\left(cation\right)}^{+}$) of ORBI molecules in solution. Thus, the lower the acid pH, the higher the molar fraction of $H_{2}ORBI_{\left(cation\right)}^{+}$ and consequently the higher the ORBI solubility, whatever the solid form in equilibrium.

In all media for which the equilibrium final pH is greater than pKa1 (except to 0.01 mol·L^−1^ HCl medium), both ORBI anhydrous and hemihydrate forms are classified as slightly soluble (Table 3) by USP criteria [9]. This means that 1 g of API is soluble in 100 to 1000 mL of solvent. As discussed above, the low solubility in this neutral media is due to the prevalence of the zwitterionic species in the equilibrium solution. In the 0.01 mol·L^−1^ HCl medium, for which the final pH was increased to ~6 during the solubility experiment, the hemihydrate form was classified as slightly soluble (1 g of API is soluble in 30 to 100 mL of test solvent), whereas the anhydrous form was sparingly soluble. The increased final pH of the medium using 0.01 mol·L^−1^ HCl can be attributed to the ORBI buffering behaviour involving the $H_{2}ORBI_{\left(cation\right)}^{+}$ and $H_{1}ORBI_{\left(\text{zwitterion}\right)}^{0,+/-}$ species released from the solid forms, which was strong enough to neutralize the introduced HCl solution. In the two media for which the equilibrium final pH is lower than pKa1 (buffer pH = 4.5 (final pH = 4.5) and 0.1 mol·L^−1^ HCl (final pH = 2)), both the ORBI anhydrous and hemihydrate forms are classified as sparingly soluble (Table 3).

A remarkable result is that the anhydrous form is significantly more soluble than the hemihydrate in all acidic media (final pH ≤ 6). The largest percentage difference (64%) occurs in 0.01 mol·L^−1^ HCl medium, which makes the solubility classification slightly soluble and sparingly soluble for the ORBI hemihydrate and anhydrous forms, respectively.

**Figure 12:**
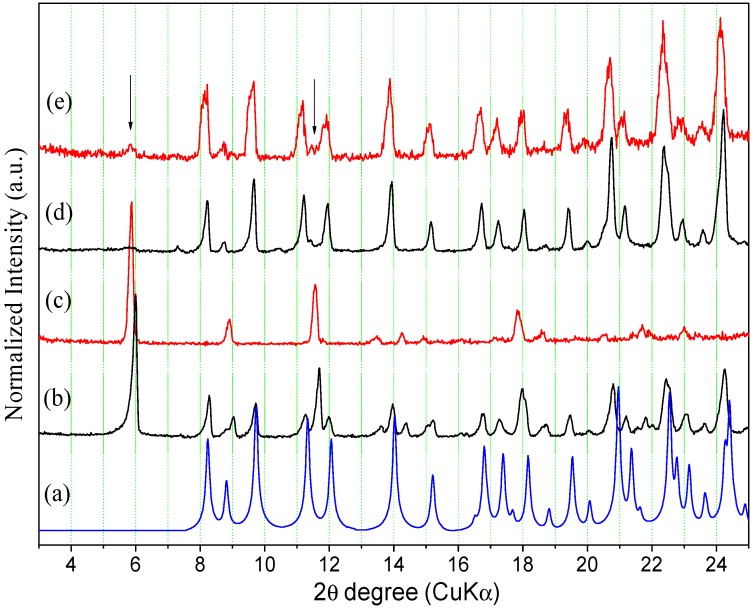
Calculated and experimental PXRD patterns of ORBI forms after the equilibrium solubility test: (a) ORBI hemihydrate form (calculated); (b) ORBI hemihydrate in 0.1 mol·L^−1^ HCl; (c) ORBI anhydrous in 0.1 mol·L^−1^ HCL; (d) ORBI hemihydrate in 0.01 mol·L^−1^ HCl (e) ORBI anhydrous in 0.01 mol·L^−1^ HCl. The extra Bragg peaks are shown by arrows.

**Figure 13:**
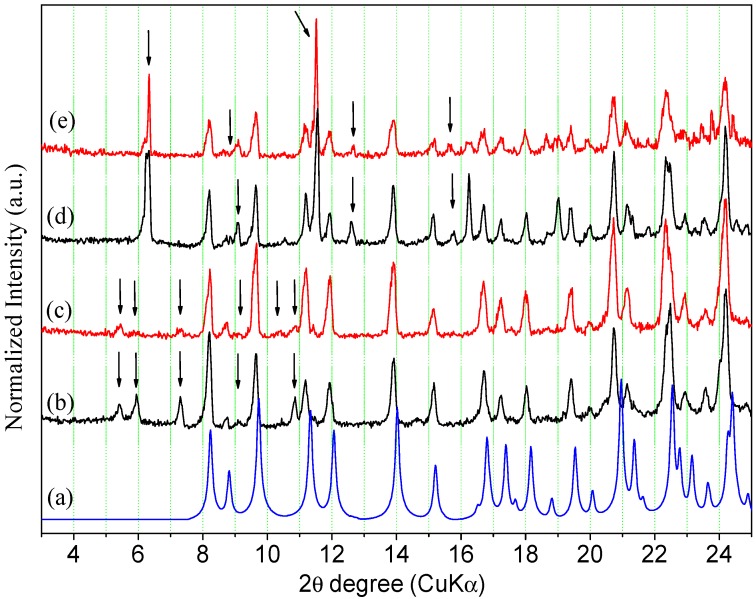
Calculated and experimental PXRD patterns of ORBI forms after the equilibrium solubility test: (a) ORBI hemihydrate form (calculated); (b) ORBI hemihydrate in pH 4.5 acetate buffer; (c) ORBI anhydrous in pH 4.5 acetate buffer (d) ORBI hemihydrate in pH 5.8 phosphate buffer (e) ORBI anhydrous in pH 5.8 phosphate buffer. The extra Bragg peaks are shown by arrows.

The highest solubility values observed for the anhydrous form compared to the hemihydrate in the acid media tested here could be attributed to the structural features of the two compared phases. Considering that hemihydrate and anhydrous forms have the same structure, the latter is expected to present a higher solubility since it is more porous and has a lower lattice energy. However, this comparison is valid only when there is no conversion of the solid form in equilibrium with the solution to a more thermodynamically stable one during the experiment. Because it was shown in Section 2.3 that the anhydrous form can be easily converted to the hemihydrate under wet conditions, the solid material in equilibrium with the solutions used to determine the ORBI equilibrium solubility was subsequently dried in a desiccator containing silica for 5 days and analysed by PXRD (Figure 12 and Figure 13, and the Figure S4 in the Supplementary Materials).

All experimental PXRD patterns of the residual solid materials in equilibrium with the solution prepared from the ORBI hemihydrate and anhydrous forms in water and in the pH 6.8 and 7.5 buffer media match the calculated PXRD pattern of the crystal structure determined here for ORBI hemihydrate (Figure S4—Supplementary Materials). Moreover, there is no apparent difference in terms of peak width of hkl indices with h ≠ 0 when the final solid materials starting from the hemihydrate and anhydrous forms are compared. Therefore, it can be concluded that the anhydrous form converts into the hemihydrate one in these three dissolution media, and consequently the equilibrium solubility values obtained for the ORBI anhydrous form could not be considered actual values.

The Bragg peaks of the ORBI hemihydrate phase (Figure 12a) do not match the experimental ones present in the PXRD pattern of the solid material separated from the ORBI anhydrous form in the 0.1 mol·L^−1^ HCl medium (Figure 12c). In other words, the PXRD analysis shows that the ORBI anhydrous form is converted to another form during the solubility determination experiment in 0.1 mol·L^−1^ HCl. The new phase is quite likely an ORBI chloride salt. This assumption is based on the fact that $H_{2}ORBI_{\left(cation\right)}^{+}$ is the unique aqueous ORBI species expected to be present in solution at pH < 3 (Figure 10), thus enabling its crystallization with the chloride from the HCl medium. However, the experimental PXRD pattern of the residual material from the hemihydrate in 0.1 mol·L^−1^ HCl medium (Figure 12b) shows Bragg peaks from the ORBI hemihydrate phase (Figure 12a) in addition to those attributed to the ORBI chloride salt (Figure 12c). Therefore, the solid material in equilibrium is a solid-state mixture containing the ORBI hemihydrate form and the probable ORBI chloride salt form crystallized during the experiment. This result suggests that the hemihydrate form is more resistant than the anhydrous one to conversion to the ORBI salt chloride form. This trend is confirmed when the experimental PXRD patterns of the residual material separated from the 0.01 mol·L^−1^ HCl media containing the ORBI hemihydrate and anhydrous forms are compared. The former PXRD pattern (Figure 12d) contains only the Bragg peaks of the hemihydrate form, whereas the latter (Figure 12e) indicates the presence of spurious Bragg peaks of very low intensity from the ORBI chloride salt form. Therefore, the percentage of conversion of the starting ORBI solid material to the ORBI chloride salt form at the end of the solubility determination experiments depends on at least two variables: (i) the pH of the aqueous media; and (ii) the crystal form used as starting material. Conversion is favoured by the use of the anhydrous form as starting material at low pH. Therefore, in spite of the observed phase transition observed in the solubility determination experiment in aqueous HCl media, which precludes the measurement of actual solubility values either for the hemihydrate or anhydrous form, the observed difference can be indirectly correlated with the stability of the two ORBI crystal forms. In other words, the anhydrous form is more unstable and consequently more susceptible to dissolution or concomitant dissolution/solid-state transformation than the hemihydrate form. The higher reactivity of the anhydrous form can be explained by the loss of crystallinity along the *a* unit cell axis (as discussed in Section 3.3) and/or by its supersaturation with respect to the hemihydrate form due to nucleation and/or crystal growth during inter-conversion in an aqueous medium [21,41].

The calculated Bragg peaks of the ORBI hemihydrate form (Figure 13a) agree with the experimental PXRD patterns of the residual solid phase in equilibrium with the pH = 4.5 acetate buffer and pH = 5.8 phosphate buffer. However, all the spectra show spurious peaks indicating the partial conversion of the initial solid phase to another one. The new phases are probably the ORBI acetate (Figure 1b,c and phosphate (Figure 13d,e) salt (see discussion above proposing the formation of the ORBI chloride salt).

## 3. Materials and Methods

### 3.1. Chemicals

Orbifloxacin standard (purity 99.9%) was purchased from Sigma-Aldrich (Seelze, Germany), and Orbifloxacin raw material (purity 99.8%) was from LKT Laboratories, Inc. (St. Paul, MN, USA). Analytical grade sodium hydroxide and hydrochloric acid were purchased from Vetec^®^ (Rio de Janeiro, Brazil), and potassium phosphate monobasic, glacial acetic acid, sodium phosphate and sodium acetate were from Proquímios^®^ (São Paulo, Brazil). Spectroscopy/HPLC grade methanol was purchased from JT Baker^®^ (Xalostoc, Mexico). Ultrapure water was obtained by reverse osmosis and filtration through a water-direct Q™ system (Millipore, Bedford, MA, USA).

Tablets of ORBI, ORBAX™ (Schering-Plough Animal Health Ind. Com. Ltda., São Paulo, Brazil), with a declared dosage of 22.7 mg API, were purchased at a local pharmacy. The excipients (mixture of inactive pharmaceutical ingredients) used in the PXRD analysis to facilitate the ORBI identification in the ORBAX™ tablets was prepared with the following ingredients: lactose monohydrate (91%) povidone (1.5%), starch glycolate sodium (6.0%), magnesium stearate (1.0%), and silicon dioxide (0.5%), which were purchased from local pharmacies.

The excipients composition lacked only the Opadry green dye, carnauba wax, and the ORBI compared with the ORBAX™ reference product. The total mass of the excipients in the formulation was obtained by determining the weight of the ORBAX™ 22.7 mg tablets (*n* = 20), and the amount of each excipient (except the lactose) was determined according to the average proportion of each component used in the production of tablets in general [42]. The amount of lactose monohydrate, which functions as a diluent/filler, was calculated by subtracting the amounts of other components from 100%.

### 3.2. Crystal Preparation

ORBI crystals were obtained by a slow solvent evaporation technique; approximately 10 mg of ORBI raw material was dissolved in approximately 50 mL of a solution of ethanol–chloroform (1:1 *v*/*v*). After five days at room temperature (25 °C) and protected from light, well-shaped white single crystals were formed. Crystals were separated from the solution for the single-crystal X-ray diffraction study.

### 3.3. Structural Determination by X-ray Diffraction for Single Crystals

The single-crystal X-ray diffraction (SCXRD) measurement was performed at low temperature (150 K) on a Gemini A-Ultra diffractometer (Oxford Diffraction, Blacksburg, VA, USA) equipped with an Atlas CCD detector using graphite-monochromatized Mo or Cu Kα beams. The programs CrysAlis CCD and CrysAlis RED [43] were used for data collection, cell refinement, data reduction, and multi-scan method absorption correction. The structure was solved using the direct method of structure factors phase retrieval using the software SHELXS-2013 [44] and refined by a full-matrix least squares on F^2^ using the software SHELXL-2013 [44]. All non-hydrogen atoms were found from the electronic density map constructed by Fourier synthesis and refined with anisotropic thermal parameters. The hydrogen atoms bonded to carbons were stereochemically positioned following a riding model with fixed C—H bond lengths of 0.93, 0.96, 0.97, and 0.98 Å for the aromatic, methyl, methylene, and methine groups, respectively. The hydrogen atoms of the water molecule and the secondary amine were refined freely after assignment using the difference Fourier map. The isotropic thermal parameters of all hydrogens depended on the equivalent isotropic thermal displacements of the atoms bonded to them [Uiso(H) = 1.2Ueq(*N*-amine, *C*-aromatic, *C*-methylene, *C*-methine) or 1.5Ueq(*O*-water, *C*-methyl)]. The zwitterionic feature of ORBI (carboxyl deprotonated and secondary amine protonated) was unambiguously determined from the electronic density map constructed by Fourier synthesis. The software WinGX [45] was used to treat the crystallographic data and generate tables. The MERCURY [46] and ORTEP-3 [47] software programs were used for the crystallographic analysis and artwork representations. CCDC 1447435 contains the supplementary crystallographic data for the ORBI structural analysis. These data have been deposited at the Cambridge Crystallographic Data Centre [35] and can be obtained free of charge via http://www.ccdc.cam.ac.uk/conts/retrieving.html (or from the CCDC, 12 Union Road, Cambridge CB2 1EZ, UK; Fax: +44 1223 336033; E-mail: deposit@ccdc.cam.ac.uk).

### 3.4. Powder X-ray Diffraction Analysis

Powder X-ray diffraction (PXRD) data were recorded at room temperature (293 K) using a Ultima IV diffractometer (Rigaku, Tokyo, Japan) with θ–2θ geometry. CuKα radiation (λ = 1.5418 Å) was generated using a sealed tube at 40 kV and 30 mA. The data were collected with step size of 0.02°. The speed of scan was 1° 2θ per minute from 3 to 35° in 2θ. The samples were finely ground and mounted on a grooved glass slide employed as a sample holder. The experimental PXRD patterns were compared with the one calculated by importing the crystal structure determined here for ORBI using single-crystal X-ray diffraction (SCXRD) data into MERCURY. The PXRD pattern calculated from low-temperature crystal structures (150 K, see Table 1) is, as expected, slightly shifted (right direction) from the experimental patterns performed at room temperature (298 K).

### 3.5. Infrared Spectroscopy with Attenuated Total Reflectance by Fourier Transform (FTIR-ATR)

FTIR-ATR spectra were obtained using an Affinity-1 Fourier transform infrared spectrophotometer (Shimadzu™, Tokyo, Japan) coupled to a Pike Miracle™ attenuated total reflectance sampling accessory with ZnSe waveguides (Pike Technologies™, Madison, WI, USA). Spectra were recorded at room temperature in the 4000–600 cm^−1^ range. After recording a background spectrum, the sample was placed on the ZnSe crystal, and 32 scans were recorded with a resolution of 2 cm^−1^.

### 3.6. Thermal Analysis

TG and DTA curves were obtained by simultaneous thermal analysis module SDT-Q-600, manufactured by TA Instruments (New Castle, DE, USA). The system was calibrated against the weight and temperature using high-purity indium, and the DTA baseline was performed and verified before analysis. The analysis were permormed using open aluminium crucibles, a heating rate of 10 °C·min**^−^**^1^, nitrogen flow at 100 mL·min**^−^**^1^, and samples of approximately 8 mg.

### 3.7. Interconversion Study between ORBI Anhydrous and Hemihydrate Forms

Approximately 50 mg of ORBI hemihydrate form (Sigma ORBI standard) was heated at 200 °C for 10 h to convert them to the ORBI anhydrous form. After allowing the samples to reach room temperature in a desiccator containing silica, complete water release from the sample was confirmed by TG measurements, which did not show mass loss in the range observed for the ORBI hemihydrate form. The dehydrated sample was studied by PXRD.

To verify the stability of the anhydrous form to humidity, 50 mg of Sigma ORBI standard previously heated at 200 °C for 10 h was placed in a climatic chamber at 40 °C with 75% of relative humidity for 1 h. Thereafter, the sample was submitted to thermogravimetric and PXRD analysis.

### 3.8. ORBI Quantification

ORBI quantification in the solubility test was performed by a stability-indicating method with high performance liquid chromatography as described by Casedey and collaborators [48]. The chromatographic separation was achieved using a Prominence model Shimadzu™ liquid chromatograph (Kyoto, Japan) with a DGU-20A 3R degasser, SIL-20AC HT autosampler, CTO-20A oven, LC-20AD pump, and SPD-M20A diode array detector. The following chromatographic parameters were used. A Shim-pack CLC-ODS Shimadzu column (Kyoto, Japan) (dimensions 250 mm × 4.6 mm diameter and 5.0 µm particle size) at 25 °C was the stationary phase. The mobile phase used was a solution of 5% (*v*/*v*) acetic acid:methanol (80:20 *v*/*v*) at a flow rate of 0.7 mL·min**^−^**^1^. UV detection was performed at a wavelength of 290 nm, and the injection volume of the sample in the chromatographic system was 20 μL.

A calibration curve was prepared using a stock solution in the mobile phase of ORBI Sigma standard (previously dried for 4 h at 105 °C in an oven) at 5 concentrations in triplicate (5, 10, 15, 20, and 30 µg·mL**^−^**^1^). The results were analysed by linear regression of the peak area *versus* concentration. The regression equation for the calibration curve was y = 134.377x − 3751 with a correlation coefficient of (*r*) = 0.9999.

### 3.9. Solubility Studies

The solubility at equilibrium was determined by the classical saturation shake-flask method [49]. The ORBI hemihydrate and anhydrous (obtained by heating the hemihydrate form at 200 °C) forms were added in excess to 2-mL Eppendorf tubes containing 1 mL of the following media: ultrapure water, hydrochloric acid 0.01 mol·L**^−^**^1^, hydrochloric acid 0.1 mol·L**^−^**^1^, pH 4.5 acetate buffer (0.050 mol·L**^−^**^1^), pH 5.8 potassium phosphate buffer (0.054 mol·L**^−^**^1^), pH 6.8 potassium phosphate buffer (0.072 mol·L**^−^**^1^), and pH 7.5 potassium phosphate buffer (0.088 mol·L**^−^**^1^).

The samples in each medium were prepared in triplicate. Subsequently, the tubes were shaken at 150 rpm on a SOLAB^TM^ SL DT 180 (São Paulo, Brazil) shaker table at room temperature (25 °C) and protected from light. After 48 h of stirring, the samples were filtered on modified PTFE filter (13-mm diameter, porosity of 0.50 µm, Advantec™ MSF, Dublin, CA, USA) and diluted in the mobile phase with the necessary dilutions. The concentration of ORBI in each solvent was determined by the chromatographic method (described in Section 3.8). The remaining solid was dried in a desiccator containing silica for 5 days and subsequently analysed by PXRD to verify that the resulting material was the same as the starting material.

## 4. Conclusions

A crystal structure of the antimicrobial API orbifloxacin was determined for the first time. This first form is zwitterionic and hemihydrated. A second form (anhydrous form) was obtained by heating the hemihydrate form. The phase transition occurs at 104 °C and the anhydrous form is stable up to 269 °C. The anhydrous form can be returned to the hemihydrate one under wet conditions. The crystal structure determined for the hemihydrate form does not collapse on water removal. Therefore, the hemihydrate and anhydrous forms of ORBI are isomorphous crystals. In spite of their isostructurally, the anhydrous form is more porous and less stable (lower lattice energy) than the hemihydrate one and consequently the former is expected to be more soluble than the later. Confirming our expectation, the lower solubility of the hemihydrate form in comparison with the anhydrous one, leads us to infer that the hemihydrate form is the one described in British Pharmacopoeia (2011) and in the USP 38 (2015), although it is presented as a dry form in these official compendia.

## Figures and Tables

**Table 1:** Crystal data and the structures refinement for ORBI hemihydrate form.

Parameter	Value
Empirical Formula	C_19_H_20_F_3_N_3_O_3_. 1/2H_2_O
Formula Weight (g∙mol^−1^)	404.4
Temperature (K)	150 (2)
Wavelength (Å)	0.71073
Crystal System	Orthorhombic
Space group	C222_1_
Cell parameters (Å, °)	a = 8.4713(4); b = 20.0456(7); c = 21.4550(7); α = β = γ = 90
Cell Volume (Å^3^)	3643.3(2)
Z; Calc. density (g∙cm^−3^)	8; 1.474
Absorption coefficient (mm^−1^)	0.123
F(000)	1688
Crystal size (mm^3^)	0.25 × 0.15 × 0.10
θ-range (°)	1.90 a 29.49
Index ranges	−11≤ *h* ≤ 11; −27 ≤ *k* ≤ 25; −29 ≤ l ≤28
Reflexions collected/unique	22,842/4627 [R(int) = 0.0281]
Completeness to θ_max_ = 29.49 (%)	100
Refinement method	Full-matrix least-squares on F^2^
Data/restraints/parameters	4627/0/267
Goodness-of-fit on F^2^	1.041
R1/wR2 for I > 2σ(I)	R1 = 0.0357, wR2 = 0.0883
R1/wR2 for all data	R1 = 0.0428, wR2 = 0.0938
Largest diff. peak/hole (e∙Å³)	0.287 and −0.202

**Table 2:** Hydrogen bonds for ORBI hemihydrate form (Å and °), where D = donor and A = acceptor.

D-H···A	d(D-H)	d(H···A)	d(D···A)	<(D-H-A)
O5^ii^-H5^ii^···O2^ii^	0.92(8)	1.98(8)	2.854(3)	159(7)
N3-H3a···O1^i^	0.88(3)	1.86(3)	2.720(2)	164(2)
N3-H3b···O2^ii^	0.86(3)	1.94(3)	2.714(2)	150(2)
N3-H3b···O3^ii^	0.86(3)	2.35(3)	2.955(2)	128(2)
C11-H11···O2^iv^	0.98	2.42	3.382(3)	168.6

d = distance; <(D-H-A) = angle. Symmetry codes as in Figure 3 and Figure 6: ^i^ –x + 1/2, y-1/2, –z + 3/2; ^ii^ –x + 1/2, -y + 1/2, z + 1/2; ^iv^ −x, y, −z + 3/2.

**Table 3:** Solubility, classification and final pH of solutions at equilibrium after 48 h at 150 rpm at 25 °C.

Media	Hemihydrate (g·L^−1^)	SD (g·L^−1^)	Solubility *	Anhydrous (g·L^−1^)	SD (g·L^−1^)	Classification *	Anhydrous/ Hemihydrate (%)	Final pH ^†^
Water	1.32	0.0678	SS	1.38	0.06634	SS	4.38	7.0
pH 7.5	1.58	0.0644	SS	1.64	0.05327	SS	3.80	7.5
pH 6.8	1.52	0.039	SS	1.59	0.07758	SS	4.60	7.0
pH 5.8	1.73	0.0601	SS	1.98	0.07915	SS	14.45	6.0
HCl 0.01 mol·L^−1^	6.30	0.1739	SS	10.33	0.29854	SP	63.97	6.0
pH 4.5	10.41	0.5007	SP	14.24	0.5539	SP	36.79	4.5
HCl 0.1 mol·L^−1^	22.24	0.7506	SP	26.23	1.0623	SP	17.94	2.0

* Legend: SS = slightly soluble; SP = sparingly soluble. Source: The United States Pharmacopeia, 38 ed., 2015; ^†^ The final pH was invariant considering the two solids forms in a same media.

## Supplementary Materials

Supplementary materials can be accessed at: http://www.mdpi.com/1420-3049/21/3/328/s1.

## Abbreviations

The following abbreviations are used in this manuscript:

### Abbreviations

ORBI: orbifloxacin
API: Active Pharmaceutical Ingredient
SCXRD: Single-Crystal X-ray Diffraction
PXRD: Powder X-ray Diffraction
TG/DTA: Thermogravimetric/Differential Thermal Analysis
FTIR-ATR: Fourier transform infrared spectroscopy-Attenuated Total Rectance
