# Supplementary material for: Structure, Solubility and Stability of Orbifloxacin Crystal Forms: Hemihydrate versus Anhydrate
Source: Molecules. 2016 Mar 9;21(3):328. doi: 10.3390/molecules21030328 (PMC6273335; doi:10.3390/molecules21030328)
Supplement: Supplementary file 1 [file molecules-21-00328-s001.pdf]

Supplementary Materials: Structure, Solubility and Stability of Orbifloxacin Crystal Forms: Hemihydrate *versus* Anhydrate

Olimpia Maria Martins Santos, Jennifer Tavares Jacon Freitas, Edith Cristina Laignier Cazedey, Magali Benjamim de Araújo and Antonio Carlos Doriguetto

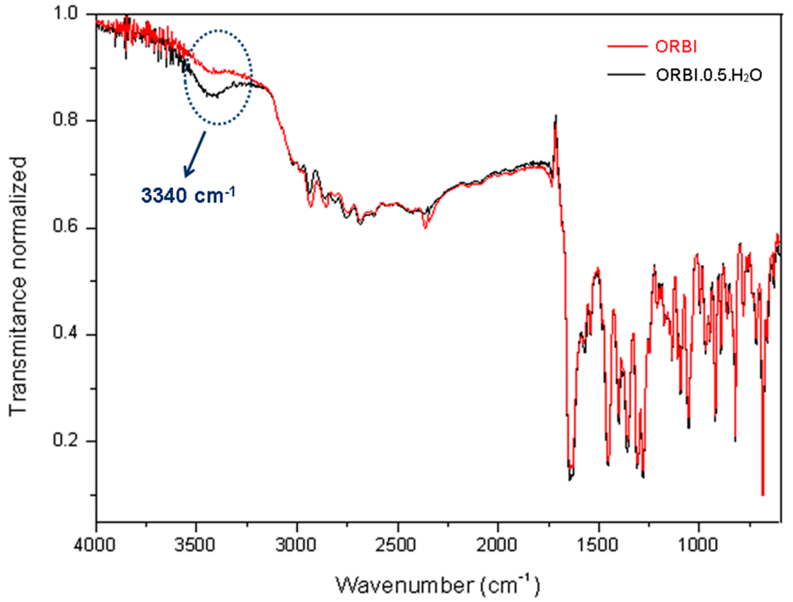

Figure S1. FTIR-ATR spectra of the ORBI hemihydrate and anhydrous forms. The band related to water molecules at 3340 cm^{-1} is highlighted.

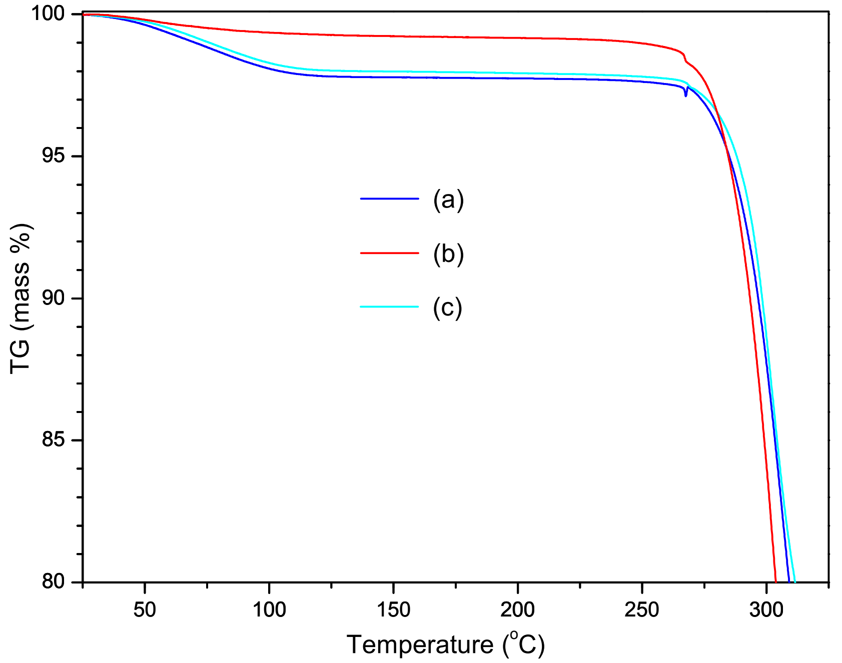

Figure S2. TG curves of (a) ORBI Sigma Standard as received (ORBI hemihydrate form); (b) ORBI Sigma Standard after heating at 200 °C for 10 h (ORBI anhydrous form); (c) ORBI Sigma Standard after heating at 200 °C for 10 h followed by treatment in climatic chamber at 40 °C with 75% relative humidity during 1 h (rehydrated form = ORBI hemihydrate Form).

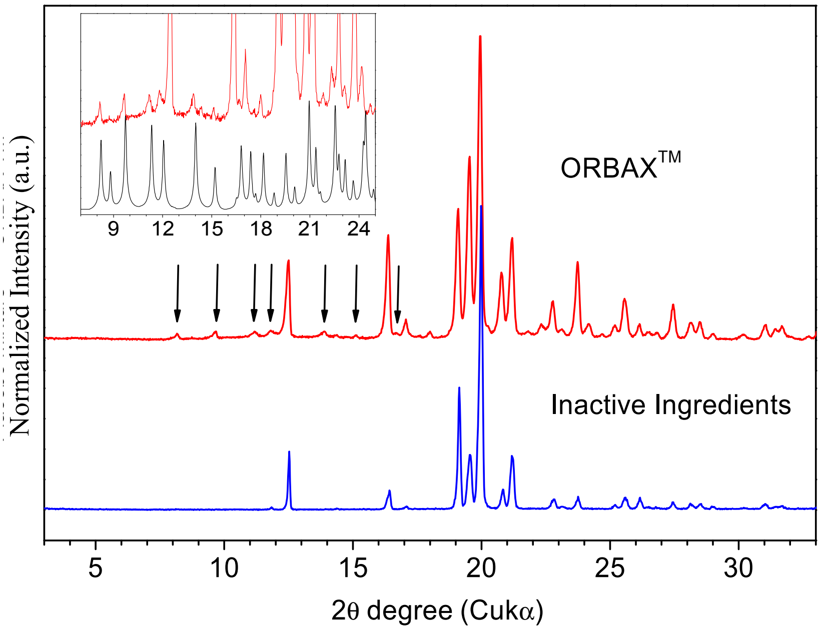

Figure S3. Experimental PXRD patterns of the inactive ingredients or excipients (in red) and ORBAX™ tablets (in black) with declared dosage of 22.7 mg of ORBI. The inset is a zoom in the 2θ range of 8–25° comparing the ORBAX™ experimental PXRD pattern with the calculated pattern of the ORBI hemihydrate form.

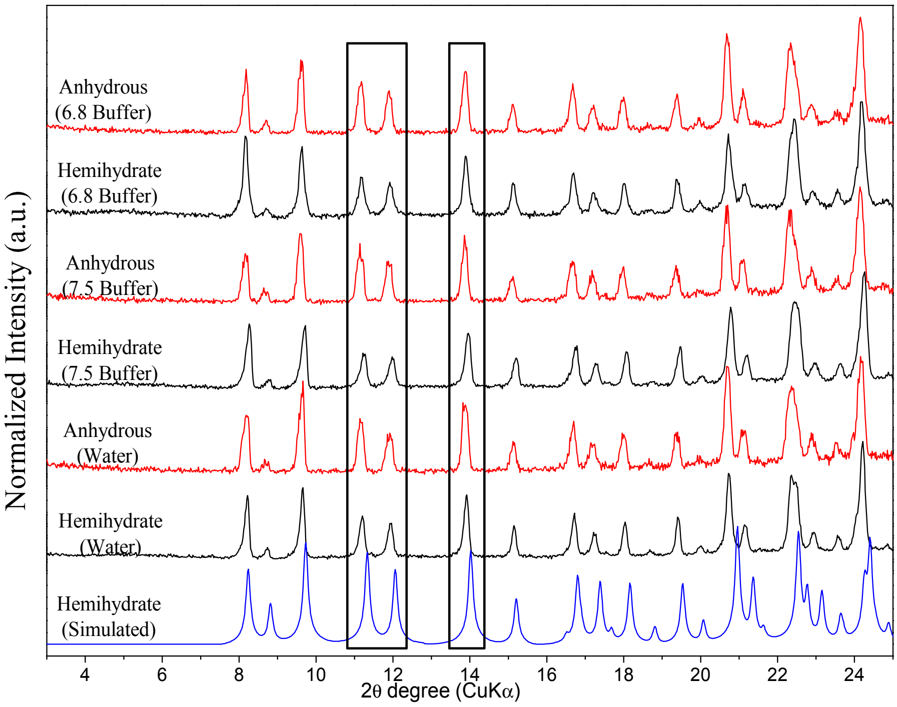

Figure S4. Experimental PXRD patterns of the residual solid materials in equilibrium with the solution prepared from the ORBI hemihydrate and anhydrous forms in water and in the pH 6.8 and 7.5 buffer media. The calculated PXRD pattern of the ORBI hemihydrate form is included.
